# Digital food delivery services in an emerging market: socioeconomic determinants and implications for service adoption in Türkiye

**DOI:** 10.1371/journal.pone.0349630

**Published:** 2026-11-12

**Authors:** Şeyda Ünver, Ahmet Kamil Kabakuş, Ömer Alkan

**Affiliations:** 1 Department of Econometrics, Ataturk University, Erzurum, Türkiye; 2 Department of Management Information Systems, Ataturk University, Erzurum, Türkiye; 3 Master Araştırma Eğitim ve Danışmanlık Hizmetleri Ltd. Şti., Ata Teknokent, Erzurum, Türkiye; Ankara University: Ankara Universitesi, TÜRKIYE

## Abstract

This study aims to identify the demographic, economic, and technological factors associated with individuals’ likelihood of ordering food online in Türkiye. Understanding these determinants is essential for enhancing digital food service accessibility and consumer satisfaction in emerging economies. Using nationally representative microdata from the 2023 Turkish Statistical Institute’s Household Information Technologies Survey, a binary logistic regression model was employed to estimate the probability of online food ordering. The model incorporated demographic variables (age, gender, education), economic factors (income and occupation), digital access indicators (computer ownership, social media use, and online information search), and regional classifications. Survey weights were applied to ensure representativeness. Results reveal that income level, age, gender, education, occupation, ownership of digital devices, social media use, and region were significantly associated with online food ordering behaviour. Women and younger, higher-income, and better-educated individuals are more likely to order food online. Regional disparities persist, with western Türkiye showing higher adoption rates than central and eastern regions, reflecting a digital divide in food delivery engagement. The findings suggest that improving technological infrastructure, digital literacy, and service quality in less developed regions could enhance inclusivity in online food delivery markets. Businesses can tailor digital marketing strategies toward younger and higher-income consumers. This study addresses an important gap in the literature by providing nationally representative evidence on the demographic, economic, and technological determinants of online food ordering behaviour in Türkiye.

## Introduction

One of the most important innovations of the twenty-first century, the Internet has become a vital marketing tool in the global economy [1]. As of 2024, 5.52 billion people globally utilise the Internet, accounting for 67.5% of the world’s population [2].

Human life is significantly impacted by information technology. According to Akhmadi, Alfathah [3], the business sector benefits significantly from information technology. Information technology facilitates information sharing between buyers and sellers, boosts productivity, and lowers transaction costs in business operations [4]. Additionally, by providing quick access to up-to-date product and pricing information, information technology enables both customers and retailers to make informed decisions. The buying and selling of goods and services using digital platforms is made possible by e-commerce, an application of information technology in the trade sector [5]. Businesses today utilise a variety of e-commerce platforms, including social media platforms, company websites, and other online trading applications [6].

Online stores are increasingly replacing physical stores, and online shopping is being favoured over traditional in-store shopping. Consumers can meet a wide range of needs through online shopping on the Internet [7]. One of the sectors significantly impacted by this transformation, where online shopping has become widespread, is the food and beverage industry. Technological and social changes have also led to shifts in consumption behaviours. Nowadays, in addition to the extensive consumption of convenience foods, people prefer to order meals online rather than cook at home. With the growth of online food ordering, numerous online food delivery websites and applications have entered the market [8]. Consumers can search for, review, and easily order their desired meals via websites or mobile applications. In Türkiye, online food ordering first began in 2001 with yemeksepeti.com and has since expanded with platforms such as GetirYemek, TıklaGelsin, TrendyolYemek, and Zomato [9].

Online food delivery services are websites or mobile technology applications that facilitate the process of placing online food orders to be picked up from physical locations using delivery drivers [10]. They primarily operate through mobile applications and can be categorised into two groups: (1) restaurants or catering businesses that develop their own applications to manage online orders and offline delivery, and (2) third-party platforms that act as intermediaries between consumers and restaurants [11].

Online shopping enables consumers to make purchases around the clock, removing the necessity for them to travel to physical stores located in different cities or countries [12]. Unlike conventional shopping, online shopping significantly reduces the time consumers spend searching for products [13]. Moreover, online shopping incurs lower search costs, as it requires less effort to compare product prices. Consequently, online shopping facilitates easier access for consumers to gather price and product information from various sellers [14]. However, despite these advantages, some consumers remain reluctant to engage in online shopping. Seeking to avoid the potential risks associated with online shopping, these consumers perceive and prefer traditional shopping as more reliable [15].

Customers of online food delivery services are unable to view the food preparation process or the store environment, unlike those in the traditional catering business. Additionally, they rarely get direct feedback from other customers and are unable to enjoy in-store eating services [16]. There are some uncertainties because customers can only assess the finished product [17].

Several notable studies have examined how these uncertainties influence consumers’ purchasing decisions [16]. For example, a prior study examining the relationship between consumers’ purchase intentions and their attitudes toward delivery platform services found that consumers’ propensity to buy is negatively impacted by negative sentiments regarding platform services [18]. The overall quality, taste, and presentation of food have a substantial impact on consumer satisfaction, according to a study that examined the relationship between restaurant meal quality, customer happiness, and behaviour. Furthermore, women pay more attention to unfavourable evaluations, which influences their purchase intentions, according to a study that used eye tracking to examine how online product reviews impact customers’ decisions to buy [19]. Another study focusing on Chinese consumers highlighted that the industry’s rapid growth is accompanied by several crucial factors that influence consumer purchasing decisions, including prices, packaging, delivery speed, ingredient freshness, reviews, transparency in kitchen operations, and hygiene practices [20].

In recent years, the rapid growth of online food ordering has made it essential to accurately identify the primary factors that influence individuals’ behaviours and attitudes toward online food ordering. This study conducts a methodical analysis to examine how various aspects influence people’s likelihood of placing online food orders. The objective of the study is to ascertain the economic and demographic factors that influence whether individuals in Türkiye have utilised a website or mobile application to place online food orders in the last three months.

## Literature review

Particularly since the COVID-19 epidemic, the use of online food ordering has drastically changed how consumers make purchases [21]. A prior study found that this change can be attributed to the efficiency and convenience offered by food aggregators, in addition to factors such as the expanding working population, increasing disposable incomes, the ease of digital payments, and the widespread use of mobile technology [22].

Restaurants have historically provided food delivery to designated areas [23]. Distribution channels have evolved in tandem with technological advancements, and online food ordering is now feasible due to the Internet [24]. When cellphones first became available, people would order food from websites. Later, both customers and businesses began using mobile food ordering apps, also known as MFOAs [25]. The literature demonstrates that a large number of studies have looked at various facets of MFOAs. According to early research on the effect of MFOAs on user preferences [26,27], users were compelled to use mobile commerce due to the features of websites and applications [23]. Another study discovered that users’ choice of application was influenced by characteristics such as real-time order tracking, secure payment choices, visual appeal, and ease of navigation [28]. According to a different study, “essential features” for mobile applications included security, design, ease, accessibility, aesthetics, and dependability [29].

However, the literature also contains several studies that examine the connection between important sociodemographic factors and online shopping through digital platforms [30]. Those who buy meals online are more likely to be young, highly educated, or have children. In contrast, older people are less likely to use these platforms, according to a cross-country study that looked at Australia, Canada, Mexico, the United Kingdom, and the United States [31]. This result was attributed to the fact that older adults are generally less tech-savvy [30]. To better understand household user behaviour, a Canadian study focusing on online prepared meal deliveries developed a demand-generating model. It indicated that characteristics such as age, income, occupation, private car ownership, and residential accessibility were significantly correlated with purchasing behaviours [32]. Another study looked at online meal delivery services in Denmark, the UK, and New Zealand. Based on the research findings, various sociodemographic factors, motivations for selecting food in e-commerce, characteristics related to the adoption of innovations, and attributes of e-service quality all significantly influence the frequency of consumers’ e-commerce food consumption [33].

Research on service quality in the context of food services is not new, as indicated by a survey of the literature [34]. Liu and Jang [35] conducted an importance–performance analysis of service quality in Chinese dining establishments, utilizing the DINESERV model. Some researchers have attempted to integrate multiple models to create a more comprehensive theoretical framework by examining service quality from various perspectives [36]. According to Markovic, Raspor [37], service quality in foodservice operations includes assessing both service results and service delivery procedures. Following the pandemic, the development of service quality in catering operations has grown more complicated due to the global emergence of online meal delivery in the catering sector. This is because a mobile application is used to physically segregate customers and catering service providers during the pre-consumption phase [38]. According to Collier and Bienstock [39], this division has a significant impact on the standards that customers use to evaluate service outcomes after consumption and to assess overall service quality perceptions during consumption. The unique role of the mobile application in the service delivery process has often been overlooked in numerous attempts to evaluate the quality of food delivery services. These attempts have primarily defined the mobile application as a service product by conflating general service quality with other service outcomes or processes [34,40].

Customer characteristics related to food delivery applications have also been studied, as noted in a review of previous research [2]. For instance, a research study conducted in South Korea utilised the Technology Acceptance Model as its theoretical framework to explore the behaviours of consumers using food delivery applications [41]. In a similar vein, the Theory of Planned Behaviour, alongside the Technology Acceptance Model, was identified as the theoretical underpinning for a food delivery application in a previous investigation carried out in Italy during the COVID-19 pandemic [8]. Moreover, findings from another study suggest that user-generated content within food delivery applications has a significant influence on consumers’ purchasing decisions [24]. Additionally, earlier research has demonstrated that the intention to purchase food delivery applications is significantly influenced by functional, social, conditional, and epistemic values [42]. The Technology Acceptance Model has also been applied in earlier studies that analysed the online food ordering behaviours of Turkish consumers [9,43,44]. Furthermore, another Turkish study that utilised the Technology Acceptance Model examined the impact of ease of use on e-satisfaction and repurchase intentions within the online meal ordering sector [2].

The Theory of Planned Behaviour (TPB) was adopted as the theoretical framework for this study [45]. According to the theory, behaviour is determined by an individual’s intention to perform a particular behaviour. Behavioural intention is influenced by three key constructs: attitudes (the perceived advantages and disadvantages of performing a behaviour), subjective norms (the social influences on behaviour), and perceived behavioural control (the facilitators and barriers to performing a behaviour) [46]. Online food ordering behaviour can be considered an example of purchasing services through the use of digital technologies. In this context, individuals’ demographic, socioeconomic, and technological characteristics are considered to be associated with online food ordering behaviour. Accordingly, the Theory of Planned Behaviour provides the conceptual basis for the selection of the independent variables and the development of the hypotheses examined in this study.

Although previous studies have investigated online food ordering from different perspectives, they have primarily focused on consumers’ behavioural intentions, technology acceptance, service quality, or specific population groups, while evidence based on nationally representative data remains limited. Therefore, a systematic analysis of the demographic, economic, and technological determinants of actual online food ordering behaviour is needed. Furthermore, Türkiye provides an important context for examining online food ordering behaviour because of its ongoing digital transformation and considerable regional socioeconomic disparities [47,48]. By using nationally representative microdata, the present study provides population-level evidence on the factors associated with actual online food ordering behaviour in an emerging digital market.

Previous studies have shown that demographic, economic, and technological characteristics are associated with individuals’ online food ordering behaviour [47,49,50]. Based on the existing literature, the following hypotheses were formulated:

**H**_**1**_: There is a significant relationship between income level and online food ordering behaviour.
**H**_**2**_: There is a significant relationship between age and online food ordering behaviour.
**H**_**3**_: There is a significant relationship between gender and online food ordering behaviour.
**H**_**4**_: There is a significant relationship between education level and online food ordering behaviour.
**H**_**5**_: There is a significant relationship between occupation and online food ordering behaviour.
**H**_**6**_: There is a significant relationship between social media use in the past three months and online food ordering behaviour.
**H**_**7**_: There is a significant relationship between online information search about goods and services in the past three months and online food ordering behaviour.
**H**_**8**_: There is a significant relationship between ownership of a desktop computer and online food ordering behaviour.
**H**_**9**_: There is a significant relationship between ownership of a laptop computer and online food ordering behaviour.
**H**_**10**_: There is a significant relationship between household size and online food ordering behaviour.
**H**_**11**_: There is a significant relationship between region and online food ordering behaviour.

## Method

### Data

Data from 8,835 respondents to the Turkish Statistical Institute’s 2023 Household Information Technologies Survey were used in this investigation. The study reflects post-pandemic consumer digitalisation patterns.

### Outcome variables

The dependent variable of the study is whether individuals placed an online food order through a website or mobile application (from restaurants, fast-food chains, or catering companies) within the last three months. Participants who had placed an online food order during the three months prior to the survey were coded as “1,” while those who had not were coded as “0”.

### Independent variables

The independent variables are: income level, age, gender, education level, occupation, social media use in the past three months, online information search about goods and services in the past three months, ownership of a desktop computer, ownership of a laptop computer, household size, and region.

Türkiye is classified into 26 NUTS Level-2 regions. In the present study, these 26 regions were grouped into three broader geographical categories (western, central, and eastern), consistent with the approach used by Ünver and Alkan (2021).

The regional classification and the provinces included in each group are presented in Table 1. The 26 NUTS Level-2 regions were consolidated into three broader regional groups to improve statistical power, reduce model complexity, and enhance the interpretability of the results.

**Table 1:** Nomenclature of Territorial Units for Statistics – Level 1 (Level 2).

Region	Code	Level 1 (Level 2)	Provinces
**Western Region**	TR1	İstanbul (TR10)	İstanbul
	TR2	West Marmara (TR21, TR22)	Tekirdağ, Edirne, Kırklareli, Balıkesir, Çanakkale
	TR3	Aegean (TR31, TR32, TR33)	İzmir, Aydın, Denizli, Muğla, Manisa, Afyonkarahisar, Kütahya, Uşak
	TR4	East Marmara (TR41, TR42)	Bursa, Eskişehir, Bilecik, Kocaeli, Sakarya, Düzce, Bolu, Yalova
**Central Region**	TR5	West Anatolia (TR51, TR52)	Ankara, Konya, Karaman
	TR6	Mediterranean (TR61, TR62, TR63)	Antalya, Isparta, Burdur, Adana, Mersin, Hatay, Kahramanmaraş, Osmaniye
	TR7	Central Anatolia (TR71, TR72)	Kırıkkale, Aksaray, Niğde, Nevşehir, Kırşehir, Kayseri, Sivas, Yozgat
	TR8	West Black Sea (TR81, TR82, TR83)	Zonguldak, Karabük, Bartın, Kastamonu, Çankırı, Sinop, Samsun, Tokat, Çorum, Amasya
**Eastern Region**	TR9	East Black Sea (TR90)	Trabzon, Ordu, Giresun, Rize, Artvin, Gümüşhane
	TRA	Northeast Anatolia (TRA1, TRA2)	Erzurum, Erzincan, Bayburt, Ağrı, Kars, Iğdır, Ardahan
	TRB	Centraleast Anatolia (TRB1, TRB2)	Malatya, Elâzığ, Bingöl, Tunceli, Van, Muş, Bitlis, Hakkâri
	TRC	Southeast Anatolia (TRC1, TRC2, TRC3)	Gaziantep, Adıyaman, Kilis, Şanlıurfa, Diyarbakır, Mardin, Batman, Şırnak, Siirt

### Statistical analysis

Complex survey design and weighting were addressed using survey statistics in Stata 17 (Stata Corporation). A weighted analysis was conducted. SPSS version 20 and Stata version 17 were utilised for data analysis. Binary logistic regression was employed to explore the connection between demographic, economic, and personal factors and online food ordering.

Binary logistic regression was preferred due to the dichotomous nature of the dependent variable and the non-linear probability structure of the relationship between online ordering behaviour and sociodemographic factors.

Logistic regression is a statistical method that allows for classification in accordance with probability rules by calculating the predicted values of the dependent variable as probabilities [51].

The logistic model was initially developed for use in survival analysis. Here, the dependent variable (Y) takes values of 1 or 0, depending on whether the event of interest occurs [52]. The expected value, E(Y), never falls below 0 or above 1. Therefore, the predicted values of $\hat{y}$ in the logistic model range between 0 and 1 [53,54].

Logistic model is written as,


$$\begin{array}{c} E(Y)=\;\pi =P(Y=\text{1})=\frac{e^{\beta_{\text{0}}+\;\beta_{\text{1}}X_{i}}}{\text{1}+\;e^{\beta_{\text{0}}+\;\beta_{\text{1}}X_{i}}}\;or\;\frac{\text{exp}(\beta_{\text{0}}+\beta_{\text{1}}X_{i})}{\text{1}+\text{exp}(\beta_{\text{0}}+\beta_{\text{1}}X_{i})}\; \end{array}$$ (1)

After dividing the numerator and denominator of Equation 1 by $e^{\beta_{\text{0}}+\;\beta_{\text{1}}X_{i}}$ or exp$\left(\beta_{\text{0}}+\beta_{\text{1}}{\text{X}}_{\text{i}}\right)$,


$$\begin{array}{c} E(Y)=\;\pi =\frac{\text{1}}{\text{1}+e^{-\left(\beta_{\text{0}}+\;\beta_{\text{1}}X_{i}\right)}}\;or\;\frac{\text{1}}{\text{1}+\text{exp}({-\beta }_{\text{0}}-\beta_{\text{1}}X_{i})} \end{array}$$ (2)

In the equation, there is a condition that $Y=\left\{ \begin{array}{cc} \text{1},\; & if\;event\;A\;occurs\\ \text{0},\; & if\;event\;B\;occurs \end{array}\right.\;$ and X values are qualitative or quantitative independent variables.

The coefficients estimated in the binary logistic regression model represent the change in the log-odds of the outcome associated with a one-unit change in the independent variable, holding the other variables constant. The sign of the coefficient indicates the direction of the association, whereas its magnitude is not directly interpretable as a change in probability.

The marginal effect is the partial derivative of the regression equation with respect to each independent variable. In the linear regression model, the slope coefficient equals the marginal effect for each variable. It represents the change in the dependent variable in response to a unit change in the independent variable. In nonlinear regression models such as the probit model, the slope is not constant across all points; it changes with the observation points, that is, the marginal effect varies with the observation points. In nonlinear regression models, the average marginal effects are interpreted, since interpretations based on any single point are not meaningful [55].

Marginal effects can be calculated in two different ways: marginal effects for means and average marginal effects. When calculating marginal effects for the means, the mean of each variable is first computed, and the marginal effects are then obtained for these means. Since the mean for dummy variables is statistically invalid, the marginal effects for the means are not appropriate in models with dummy variables among the independent variables. When calculating the average marginal effects, marginal effects are calculated for all units in the data set, that is, for each observation point, and these marginal effects are averaged. Since the models used contain categorical independent variables, average marginal effects were calculated [55].

Marginal effects (ME) were preferred for interpretation because they provide more intuitive probability-based interpretations than logistic regression coefficients or odds ratios, particularly in models containing numerous categorical variables.

### Ethics statement

We declare that all ethical guidelines for authors have been followed by all authors. Ethical approval is not required [56].

TurkStat is an institution that compiles, evaluates, and presents statistical information to decision-makers to prepare development plans and programs, make economic decisions, and address all other issues needed. TurkStat carries out internationally comparable statistical production activities according to the standards of organizations such as the European Union Statistical Office, the United Nations, OECD, ILO, etc. TurkStat collects data within the scope of the Official Statistics Program. The Official Statistics Program is prepared for five-year periods based on the Turkish Statistics Law No. 5429 to determine the basic principles and standards regarding the production and publication of official statistics and to ensure the production of up-to-date, reliable, timely, transparent and impartial data in areas of need at national and international levels [56]. The individuals included in the sample in the surveys conducted by the Turkish Statistical Institute are legally obliged to participate in the survey. According to the Turkish Statistics Law, those who do not provide the information requested within the scope of the research in the specified form and time without a valid excuse or who provide incomplete or incorrect information are warned once and asked to provide the information or correct the deficiencies and errors within seven days. An administrative fine shall be imposed on natural persons or organs and representatives of private legal entities who, despite this warning, do not provide the information or correct the deficiencies and errors as requested [56]. TurkStat also conducts the Household Information Technologies Usage Survey within the scope of the Official Statistics Program put into effect by law. Since the Household Information Technologies Usage Survey Microdata Set is conducted within the scope of legal responsibility by the state, ethical approval is not required [57].

The data were obtained through the joint teamwork of both the Turkish Statistical Institute (TurkStat) and the European Union Statistical Office (SOEU). We obtained this data from TurkStat in return for a contract without needing an ethics committee document and used it in our study [56].

## Results

### Findings regarding variables

Among individuals aged 16–24 years, 45.4% had ordered food online within the previous three months. Among high-school graduates, 43.1% had ordered food online. Among laptop owners, 50.8% had ordered food online. The findings regarding factors associated with individuals’ online food ordering behaviour in Türkiye are presented in Table 2.

**Table 2:** Findings regarding factors related to online food ordering.

Variables		Online Food Ordering Status			
		No		Yes	
		n	%	n	%
**Gender**	Female	2,556	57.5	1,887	42.5
	Male	2,426	55.2	1,966	44.8
**Age**	16-24	1,213	54.6	1,010	45.4
	25-34	1,455	50.6	1,421	49.4
	35-44	1,330	60.0	887	40.0
	45-54	676	61.4	425	38.6
	55-64	235	71.9	92	28.1
	65+	73	80.2	18	19.8
**Level of education**	No formal education	33	82.5	7	17.5
	Primary school	528	76.3	164	23.7
	Middle school	881	67.8	419	32.2
	High school	1,709	56.9	1,292	43.1
	University	1,831	48.2	1,971	51.8
**Income level**	1^st^ income level (lowest))	496	72.5	188	27.5
	2^nd^ income level	866	66.8	431	33.2
	3^rd^ income level	1,044	59.6	707	40.4
	4^th^ income level	1,208	55.6	964	44.4
	5^th^ income level (highest)	1,368	46.7	1,563	53.3
**Desktop computer ownership**	No	4,016	57.6	2,954	42.4
	Yes	966	51.8	899	48.2
**Laptop ownership**	No	2,576	65.3	1,369	34.7
	Yes	2,406	49.2	2,484	50.8
**Region**	West	1,893	47.0	2,135	53
	Central	1,647	57.9	1,197	42.1
	East	1,442	73.5	521	26.5
**Occupation**	Managers	241	46.5	277	53.5
	Professionals	636	43.0	844	57
	Technicians/associate professionals	271	47.4	301	52.6
	Clerical support workers	232	46.2	270	53.8
	Service/sales workers	462	54.9	380	45.1
	Skilled agricultural/forestry/fishery workers	56	83.6	11	16.4
	Craft and related trades workers	269	65.5	142	34.5
	Plant/machine operators/assemblers	243	63.6	139	36.4
	Elementary occupations	293	72.0	114	28
	Unemployed	2,279	62.4	1,375	37.6
**Social media use**	No	302	66.8	150	33.2
	Yes	4,680	55.8	3,703	44.2
**Information search about goods/services**	No	5	41.7	7	58.3
	Yes	4,977	56.4	3,846	43.6
**Household size**	1-3	2,154	51.0	2,073	49.0
	4-5	2,285	59.5	1,557	40.5
	6 and above	543	70.9	223	29.1

### Model estimation

Model fit was evaluated using likelihood ratio statistics and pseudo R² measures. The overall model was statistically significant (LR χ²(31) = 1200.98, p < 0.001), indicating that the predictors jointly improved the model compared with the intercept-only model. McFadden’s pseudo R² was 0.099, while the Cragg and Uhler (Nagelkerke) R² was 0.127. The model correctly classified 65.9% of the observations. Multicollinearity among the independent variables was assessed using the Variance Inflation Factor (VIF) and Tolerance (1/VIF) statistics. All VIF values were below 5, tolerance values exceeded 0.20, and the mean VIF was 1.55, indicating that multicollinearity was not a concern. Table 3 presents the estimated regression coefficients (β), Table 4 presents the corresponding odds ratios (ORs) with 95% confidence intervals, and Table 5 reports the marginal effects of the explanatory variables. Overall, income level, age, gender, education level, occupation, desktop computer ownership, laptop ownership, social media use, information search for goods/services, household size, and region were significantly associated with online food ordering behaviour.

**Table 3:** Estimation results for coefficients.

Variables					Confidence Interval		
		β	S.E	z	Lower limit	Upper limit	VIF
**Gender (Reference: male)**							
	Female	0.162^a^	0.051	3.19	0.063	0.262	1.24
**Age (Reference: 16–24)**							
	25-34	−0.190^a^	0.068	−2.81	−0.322	−0.057	1.91
	35-44	−0.572^a^	0.072	−7.91	−0.714	−0.431	1.84
	45-54	−0.786^a^	0.087	−9.03	−0.957	−0.615	1.55
	55-64	−1.310^a^	0.142	−9.25	−1.588	−1.033	1.20
	65+	−1.871^a^	0.280	−6.69	−2.419	−1.322	1.07
**Level of education (Reference: University)**							
	No formal education	−0.724	0.453	−1.60	−1.611	0.163	1.03
	Primary school	−0.448^a^	0.110	−4.07	−0.663	−0.232	1.43
	Middle school	−0.467^a^	0.083	−5.64	−0.629	−0.305	1.59
	High school	−0.064	0.062	−1.04	−0.185	0.057	1.67
**Income level (Reference: 1**^**st**^ **income level (lowest))**							
	2^nd^ income level	0.269^b^	0.111	2.42	0.051	0.487	2.53
	3^rd^ income level	0.442^a^	0.106	4.18	0.235	0.650	2.96
	4^th^ income level	0.463^a^	0.105	4.42	0.258	0.669	3.41
	5^th^ income level (highest)	0.746^a^	0.105	7.11	0.540	0.951	4.12
**Desktop computer ownership (Reference: no)**							
	Yes	0.130^b^	0.057	2.29	0.019	0.241	1.05
**Laptop ownership (Reference: no)**							
	Yes	0.331^a^	0.051	6.50	0.231	0.43	1.23
**Occupation**	**(Reference: Unemployed)**						
	Managers	0.326^a^	0.109	3.00	0.113	0.540	1.29
	Professionals	0.391^a^	0.081	4.84	0.233	0.549	1.80
	Technicians/associate professionals	0.271^a^	0.102	2.65	0.070	0.471	1.26
	Clerical support workers	0.364^a^	0.106	3.44	0.156	0.571	1.19
	Service/sales workers	0.171^b^	0.086	1.98	0.002	0.340	1.26
	Skilled agricultural/forestry/fishery workers	−0.938^a^	0.347	−2.70	−1.619	−0.257	1.03
	Craft and related trades workers	−0.197	0.121	−1.62	−0.435	0.042	1.20
	Plant/machine operators/assemblers	−0.114	0.124	−0.92	−0.357	0.129	1.19
	Elementary occupations	−0.365^a^	0.125	−2.91	−0.611	−0.119	1.15
**Region (Reference: west)**							
	Central	−0.460^a^	0.052	−8.80	−0.562	−0.357	1.18
	East	−1.142^a^	0.065	−17.65	−1.269	−1.015	1.25
**Social media use (Reference: no)**							
	Yes	0.371^a^	0.110	3.38	0.156	0.586	1.02
**Information search about goods/services (Reference: no)**							
	Yes	−1.242^c^	0.647	−1.92	−2.511	0.026	1.00
**Household size (Reference: 1–3)**							
	4-5	−0.293^a^	0.050	−5.85	−0.391	−0.195	1.19
	6 and above	−0.512^a^	0.094	−5.47	−0.695	−0.328	1.19

^a^p < .01; ^b^p < .05; ^c^p < .10

**Table 4:** Odds ratios (ORs) and 95% confidence intervals.

Variables					Confidence Interval		
		OR	S.E	z	Lower limit		Upper limit
**Gender (Reference: male)**							
	Female	1.176	0.060	3.19	1.064		1.300
**Age (Reference: 16–24)**							
	25-34	0.827	0.056	−2.81	0.725		0.944
	35-44	0.564	0.041	−7.91	0.490		0.650
	45-54	0.456	0.040	−9.03	0.384		0.541
	55-64	0.270	0.038	−9.25	0.204		0.356
	65+	0.154	0.043	−6.69	0.089		0.267
**Level of education (Reference: University)**							
	No formal education	0.485	0.219	−1.60	0.200		1.177
	Primary school	0.639	0.070	−4.07	0.515		0.793
	Middle school	0.627	0.052	−5.64	0.533		0.737
	High school	0.938	0.058	−1.04	0.831		1.058
**Income level (Reference: 1**^**st**^ **income level (lowest))**							
	2^nd^ income level	1.309	0.146	2.42	1.053		1.628
	3^rd^ income level	1.557	0.165	4.18	1.265		1.916
	4^th^ income level	1.590	0.167	4.42	1.294		1.952
	5^th^ income level (highest)	2.108	0.221	7.11	1.716		2.589
**Desktop computer ownership (Reference: no)**							
	Yes	1.139	0.065	2.29	1.019		1.273
**Laptop ownership (Reference: no)**							
	Yes	1.392	0.071	6.50	1.260		1.538
**Occupation**	**(Reference: Unemployed)**						
	Managers	1.386	0.151	3.00	1.120		1.716
	Professionals	1.478	0.119	4.84	1.262		1.713
	Technicians/associate professionals	1.311	0.134	2.65	1.073		1.602
	Clerical support workers	1.438	0.152	3.44	1.169		1.770
	Service/sales workers	1.186	0.102	1.98	1.002		1.405
	Skilled agricultural/forestry/fishery workers	0.392	0.136	−2.70	0.198		0.774
	Craft and related trades workers	0.822	0.100	−1.62	0.648		1.042
	Plant/machine operators/assemblers	0.892	0.111	−0.92	0.700		1.138
	Elementary occupations	0.694	0.087	−2.91	0.543		0.888
**Region (Reference: west)**							
	Central	0.631	0.033	−8.80	0.570		0.700
	East	0.319	0.021	−17.65	0.281		0.362
**Social media use (Reference: no)**							
	Yes	1.449	0.159	3.38	1.169		1.797
**Information search about goods/services (Reference: no)**							
	Yes	0.289	0.187	−1.92	0.081		1.027
**Household size (Reference: 1–3)**							
	4-5	0.746	0.037	−5.85	0.676		0.823
	6 and above	0.599	0.056	−5.47	0.499		0.720

**Table 5:** Average marginal effects of factors associated with individuals’ online food ordering behaviour.

Variables					Confidence Interval	
		M.E	S.E	z	Lower limit	Upper limit
**Gender (Reference: male)**						
	Female	0.035^a^	0.011	3.19	0.013	0.056
**Age (Reference: 16-24)**						
	25-34	-0.041^a^	0.015	-2.82	-0.070	-0.013
	35-44	-0.124^a^	0.015	-8.06	-0.154	-0.094
	45-54	-0.168^a^	0.018	-9.40	-0.203	-0.133
	55-64	-0.266^a^	0.025	-10.69	-0.315	-0.217
	65+	-0.350^a^	0.037	-9.46	-0.423	-0.277
**Level of education (Reference: university)**						
	No formal education	-0.152^c^	0.088	-1.73	-0.324	0.020
	Primary school	-0.097^a^	0.023	-4.14	-0.142	-0.051
	Middle school	-0.100^a^	0.018	-5.70	-0.135	-0.066
	High school	-0.014	0.014	-1.04	-0.041	0.013
**Income level (Reference: 1^st^ income level (lowest))**						
	2^nd^ income level	0.056^b^	0.023	2.45	0.011	0.100
	3^rd^ income level	0.093^a^	0.022	4.25	0.050	0.135
	4^th^ income level	0.097^a^	0.021	4.55	0.055	0.139
	5^th^ income level (highest)	0.160^a^	0.022	7.41	0.117	0.202
**Desktop computer ownership (Reference: no)**						
	Yes	0.028^b^	0.012	2.28	0.004	0.052
**Laptop ownership (Reference: no)**						
	Yes	0.072^a^	0.011	6.47	0.050	0.094
**Occupation**	**(Reference: unemployed)**					
	Managers	0.072^a^	0.024	2.97	0.025	0.119
	Professionals	0.086^a^	0.018	4.80	0.051	0.121
	Technicians/associate professionals	0.060^a^	0.023	2.63	0.015	0.104
	Clerical support workers	0.080^a^	0.024	3.41	0.034	0.126
	Service/sales workers	0.037^b^	0.019	1.97	0.000	0.075
	Skilled agricultural/forestry/fishery workers	-0.182^a^	0.057	-3.18	-0.294	-0.070
	Craft and related trades workers	-0.042	0.026	-1.64	-0.092	0.008
	Plant/machine operators/assemblers	-0.025	0.027	-0.93	-0.077	0.027
	Elementary occupations	-0.077^a^	0.026	-3.00	-0.127	-0.027
**Region (Reference: west)**						
	Central	-0.104^a^	0.012	-8.87	-0.126	-0.081
	East	-0.244^a^	0.013	-19.04	-0.270	-0.219
**Social media use (Reference: no)**						
	Yes	0.078^a^	0.022	3.48	0.034	0.122
**Information search about goods/services (Reference: no)**						
	Yes	-0.258^b^	0.120	-2.16	-0.493	-0.024
**Household size (Reference: 1-3)**						
	4-5	-0.064^a^	0.011	-5.86	-0.085	-0.042
	6 and above	-0.110^a^	0.020	-5.60	-0.148	-0.071

^a^p < .01; ^b^p < .05; ^c^p < .10

The corresponding odds ratios (ORs) and their 95% confidence intervals are presented in Table 4.

An odds ratio (OR) greater than 1 indicates higher odds of the outcome relative to the reference category, whereas an OR less than 1 indicates lower odds of the outcome. The magnitude of the OR reflects the strength of the association relative to the reference group [50]. According to Table 4, females have higher odds of ordering food online than males (OR = 1.176; 95% CI = 1.064–1.300). Compared with individuals aged 16–24 years, those aged 25–34 years (OR = 0.827; 95% CI = 0.725–0.944), 35–44 years (OR = 0.564; 95% CI = 0.490–0.650), 45–54 years (OR = 0.456; 95% CI = 0.384–0.541), 55–64 years (OR = 0.270; 95% CI = 0.204–0.356), and 65 years and above (OR = 0.154; 95% CI = 0.089–0.267) have lower odds of ordering food online. Compared with individuals with a university education, those with primary school (OR = 0.639; 95% CI = 0.515–0.793) and middle school education (OR = 0.627; 95% CI = 0.533–0.737) have lower odds of ordering food online. Compared with individuals in the lowest income level, those in the second (OR = 1.309; 95% CI = 1.053–1.628), third (OR = 1.557; 95% CI = 1.265–1.916), fourth (OR = 1.590; 95% CI = 1.294–1.952), and highest income level (OR = 2.108; 95% CI = 1.716–2.589) have higher odds of ordering food online. Individuals who own a desktop computer (OR = 1.139; 95% CI = 1.019–1.273) and a laptop computer (OR = 1.392; 95% CI = 1.260–1.538) have higher odds of ordering food online than those who do not. Compared with unemployed individuals, managers (OR = 1.386; 95% CI = 1.120–1.716), professionals (OR = 1.478; 95% CI = 1.262–1.713), technicians and associate professionals (OR = 1.311; 95% CI = 1.073–1.602), clerical support workers (OR = 1.438; 95% CI = 1.169–1.770), and service and sales workers (OR = 1.186; 95% CI = 1.002–1.405) have higher odds of ordering food online, whereas skilled agricultural, forestry and fishery workers (OR = 0.392; 95% CI = 0.198–0.774) and individuals in elementary occupations (OR = 0.694; 95% CI = 0.543–0.888) have lower odds. Compared with individuals living in the western region, those living in the central region (OR = 0.631; 95% CI = 0.570–0.700) and the eastern region (OR = 0.319; 95% CI = 0.281–0.362) have lower odds of ordering food online. Individuals who use social media have higher odds of ordering food online than those who do not (OR = 1.449; 95% CI = 1.169–1.797). Finally, compared with individuals living in households consisting of 1–3 members, those living in households with 4–5 members (OR = 0.746; 95% CI = 0.676–0.823) and six or more members (OR = 0.599; 95% CI = 0.499–0.720) have lower odds of ordering food online.

Table 5 presents the marginal effects of the variables related to people’s online food ordering behaviour.

According to the average marginal effects presented in Table 5, the predicted probability of ordering food online is 3.5 percentage points higher for females than for males. Compared with individuals aged 16–24 years, the predicted probability of ordering food online is 4.1 percentage points lower for individuals aged 25–34 years, 12.4 percentage points lower for those aged 35–44 years, 16.8 percentage points lower for those aged 45–54 years, 26.6 percentage points lower for those aged 55–64 years, and 35.0 percentage points lower for those aged 65 years and over, holding the other variables constant.

Compared with university graduates, the predicted probability of ordering food online is 15.2 percentage points lower for individuals with no formal education, 9.7 percentage points lower for primary school graduates, and 10.0 percentage points lower for middle school graduates. Compared with individuals in the 1st (lowest) income level, the predicted probability of ordering food online is 5.6 percentage points higher for individuals in the 2nd income level, 9.3 percentage points higher for those in the 3rd income level, 9.7 percentage points higher for those in the 4th income level, and 16.0 percentage points higher for those in the 5th (highest) income level, holding the other variables constant.

Compared with individuals who do not own these devices, the predicted probability of ordering food online is 2.8 percentage points higher for desktop computer owners and 7.2 percentage points higher for laptop owners. Compared with unemployed individuals, the predicted probability of ordering food online is 7.2 percentage points higher for managers, 8.6 percentage points higher for professionals, 6.0 percentage points higher for technicians and associate professionals, 8.0 percentage points higher for clerical support workers, and 3.7 percentage points higher for service and sales workers. In contrast, the predicted probability is 18.2 percentage points lower for skilled agricultural, forestry, and fishery workers and 7.7 percentage points lower for individuals in elementary occupations, holding the other variables constant.

Compared with individuals living in the western region, the predicted probability of ordering food online is 10.4 percentage points lower for individuals living in the central region and 24.4 percentage points lower for those living in the eastern region. The predicted probability of ordering food online is 7.8 percentage points higher for individuals who used social media within the last three months than for those who did not. Individuals who searched for information about goods and services on the Internet within the last three months have a predicted probability of ordering food online that is 25.8 percentage points lower than that of individuals who did not engage in this activity. Compared with individuals living in households with 1–3 members, the predicted probability of ordering food online is 6.4 percentage points lower for those living in households with 4–5 members and 11.0 percentage points lower for those living in households with six or more members, holding the other variables constant.

## Discussion and Conclusion

The purpose of this study was to identify the variables that influence the probability of people in Türkiye placing an online food order within the previous three months. Studies show that women are more likely than men to place online food orders. This finding is consistent with previous studies reporting that women are generally more active participants in e-commerce, particularly during and after the COVID-19 pandemic [58, 59]. However, not all studies have reached the same conclusion. For example, a study conducted in Türkiye found no significant gender differences in online shopping preferences for food items, daily necessities, and household goods [60], suggesting that the influence of gender may vary depending on the product category examined. One possible explanation is that Turkish society has a traditional patriarchal structure in which women are more actively involved in household food-related purchasing decisions [61]. Consequently, women may place greater value on the convenience and time-saving benefits offered by online food delivery services.

The results of this research further suggest that the likelihood of ordering food online decreases with age. A previous study suggested that as consumers grow older, online shopping requires greater physical effort [62]. Another study focusing on the Turkish context argued that fewer e-commerce experiences among older adults is an expected outcome, as keeping up with technological innovations and learning quickly slows down with age [63]. Similarly, another study highlighted that individuals in different age groups may exhibit different tendencies toward e-commerce usage [64]. One possible explanation is that older individuals may be less willing to adopt new digital technologies and may perceive online food ordering platforms as more difficult to use than younger individuals.

Furthermore, the study found that higher levels of education are associated with an increased likelihood of placing online food orders. Similar demographic findings have also been reported in earlier research [65–67]. A prior study conducted in Türkiye investigated the differences in e-commerce use by education level and found that the significance and impact of variables vary across educational backgrounds. According to that study, individuals’ propensity to engage in online shopping increases as their level of education rises [68]. One possible explanation is that individuals with higher educational attainment may have greater digital literacy and be more confident in using online platforms. Previous studies have also shown that individuals with higher levels of education are more likely to seek information on the internet, including health-related information [69]. This suggests that higher educational attainment promotes broader engagement with digital technologies. Consequently, they may be more willing to adopt and use online food ordering services.

According to the study, individuals’ likelihood of placing online food orders increases as their income level rises. This discovery aligns with earlier research, which has demonstrated that increased income is correlated with a higher likelihood of engaging in e-commerce [64, 70, 71]. Earlier research has emphasised that high-income individuals tend to possess higher qualifications and are more likely to adopt new technologies [70, 72]. Furthermore, consumers with different income levels hold varying perceptions of service quality [73], with affluent clients often pursuing superior service engagements [74]. As a result, higher-income consumers may also be more willing to pay premium prices in exchange for better service quality and convenience [75]. One possible explanation is that individuals with higher income levels can more easily afford delivery fees and other additional costs associated with online food ordering. They may also place greater value on the convenience and time-saving benefits of these services. Consequently, they may be more willing to use online food ordering services.

Online food ordering is more common among desktop computer owners than among those without one. Similarly, individuals who own a laptop are more likely to order online food than those who do not. One possible explanation is that owning a desktop computer or laptop facilitates access to online platforms and increases familiarity with digital services. Previous studies have also shown that individuals who own digital devices are more likely to use the internet for activities such as seeking health-related information [76, 77]. Consequently, they may be more willing to adopt and use online food ordering services.

With respect to occupation, individuals in the managerial category are more likely to order food online than those who are unemployed. Similarly, professionals, technicians and associate professionals, clerical support workers, and service and sales workers are more likely to order food online than unemployed individuals. In contrast, skilled agricultural, forestry, and fishery workers, as well as those engaged in elementary occupations, are less likely to order food online than unemployed individuals. This outcome can be attributed to differences in individuals’ professional experiences and variations in occupational characteristics across different societies [70, 78].

One possible explanation for these findings is that managerial and professional occupations, technicians and associate professional occupations, and clerical support occupations may be associated with heavier workloads, greater time constraints, and more frequent use of digital technologies in daily work. Consequently, individuals in these occupational groups may place greater value on the convenience and time-saving benefits of online food ordering services. The greater interaction of service and sales workers with digital systems and consumer-oriented services may also be associated with their higher likelihood of ordering food online. In contrast, skilled agricultural, forestry, and fishery workers and individuals engaged in elementary occupations may have more limited exposure to digital technologies in their work environments and different daily routines, which may be associated with their lower use of online food ordering services.

The study also found that individuals from the central region are less likely to order food online compared to those from the western region. In contrast, individuals from the eastern region are even less likely to do so. A previous study examining the regional differences in online shopping among Turkish consumers found that the significance and impact of variables related to e-commerce usage also vary by region. The results of that research suggested that enhancing e-commerce growth in underdeveloped regions necessitates the advancement of information and communication technology infrastructure, as well as encouraging wider internet access in those locations [47]. One possible explanation is that the digital divide between regions with different levels of socioeconomic development may influence the adoption and use of online food ordering services. Previous research has shown that disparities in regional development affect the use of telecommunications and other advanced technologies [79]. In addition, socioeconomic factors shape the adoption of information and communication technologies, further contributing to differences in digital service use across regions [47].

Furthermore, individuals who used social media within the last three months are more likely to place online food orders than those who did not. Alongside technology ownership, individuals’ use of digital applications is also a determinant of e-commerce participation. Considering the growing popularity of advertisements and promotional activities conducted through social media, these platforms can be regarded as an important factor influencing consumers’ inclination to order food online [80, 81]. One possible explanation is that social media increases individuals’ exposure to food-related advertisements, promotional campaigns, and recommendations. Frequent interaction with these platforms may also increase familiarity with online food delivery applications and encourage spontaneous purchasing decisions. Consequently, social media users may be more willing to order food online.

Individuals who have sought information regarding products and services online within the past three months are less likely to order food online compared to those who have not sought such information during the same period. Contrary to this study, previous research has shown that individuals who use the internet to search for information about goods and services are more likely to engage in e-commerce, and similar findings have been reported in other studies [63, 82]. One possible explanation for this inconsistency is that the variable used in the present study measures general online information searching for goods and services rather than information seeking specifically related to online food ordering. Consequently, individuals may search for information about products such as electronics, clothing, travel, or other services without subsequently ordering food online. The coefficient was marginally significant at the 10% level, whereas the corresponding average marginal effect reached the 5% significance level; therefore, the finding should be interpreted cautiously. Future studies using variables that specifically measure information seeking related to online food ordering, rather than general searches for goods and services, may help clarify this relationship.

An unexpected finding of this study was the negative association between online information search about goods and services and online food ordering behaviour. Although previous studies generally suggest that online information search facilitates digital purchasing behaviour, this finding may reflect the distinction between general information-seeking behaviour and actual purchasing behaviour. It should be noted that the TurkStat variable measures general online information searches for goods and services rather than food-related searches specifically. Therefore, individuals who search online may primarily compare prices, evaluate alternatives, or gather product information without necessarily completing an online food order. Some consumers may also use online sources to obtain information before purchasing through offline channels. From the perspective of the Theory of Planned Behaviour, information search alone may not directly translate into behavioural intention or actual purchasing behaviour because the transition from information acquisition to purchase is also influenced by attitudes, perceived behavioural control, and contextual factors. Therefore, this unexpected finding should be interpreted cautiously and warrants further investigation using more specific measures of food-related online search behaviour.

Accordingly, the observed association should not necessarily be interpreted as evidence that online information searching discourages online food ordering, but rather as reflecting the broader behavioural scope of the survey measure.

Individuals in households with a household size of 4–5 people are less likely to order food online than those in households with a household size of 1–3 people. Individuals in households with a household size of 6 or more people are less likely to order food online than those in households with a household size of 1–3 people. In a similar vein, earlier research has indicated that the probability of engaging in e-commerce diminishes with an increase in household size [47, 83]. Conversely, a prior study concluded that household size does not influence e-commerce usage [84]. One possible explanation is that larger households may prefer preparing meals at home because food can be shared among more family members, making online food ordering relatively less economical. In addition, larger households may have more diverse food preferences, making it more difficult to satisfy everyone with a single online order. Consequently, individuals living in larger households may be less likely to use online food ordering services.

The research’s conclusions can provide academics and decision-makers with important information about how to encourage online food ordering in Türkiye’s growing areas. By ensuring efficient client satisfaction through the operational efficiencies provided by online meal ordering software, this project is expected to enhance social welfare and quality of life. Additionally, people in charge of food ordering apps should pay greater attention to the calibre, cleanliness, and cost of the food they serve, in addition to giving customers more thorough details about the apps they use. Additionally, companies that offer online meal ordering want to develop plans that improve customers’ opinions about the safety and quality of the meals. On the other hand, one of the study’s recommendations is that pricing strategies be organised within a framework that is both transparent and competitive.

This study has several limitations. First, it is based on secondary data from the Turkish Statistical Institute Household Information Technologies Survey, limiting the analysis to the variables available in the dataset. Second, the cross-sectional design precludes causal inference between online food ordering behaviour and the associated socioeconomic factors. The online information-search variable was highly unbalanced, with very few respondents in the reference category; therefore, estimates for this variable should be interpreted cautiously. Finally, the findings rely on self-reported survey responses, which may be affected by reporting and recall bias. Therefore, the results should be interpreted with these limitations in mind.
