# Supplementary material for: Digital food delivery services in an emerging market: socioeconomic determinants and implications for service adoption in Türkiye
Source: PLoS One. 2026 Nov 12;21(11):e0349630. doi: 10.1371/journal.pone.0349630 (PMC13759073; doi:10.1371/journal.pone.0349630)
Supplement: Supplementary file 1 [file pone.0349630.s002.docx]

**Dear Editor and Reviewers,**

Thank you very much for your comments concerning our manuscript titled “Digital Food Delivery Services in an Emerging Market: Socioeconomic Determinants and Implications for Service Adoption in Türkiye”. These comments have been very helpful in reviewing and improving our manuscript. We have carefully revising these instructive comments and made corrections that we hope will be approved. The revised parts are highlighted in red in the main paper.

The corrections to the manuscript and responses to the reviewer’s comments are as following:

Regards,

Authors

**Journal Requirements:**

When submitting your revision, we need you to address these additional requirements.

1. Please ensure that your manuscript meets PLOS ONE's style requirements, including those for file naming. The PLOS ONE style templates can be found at

<https://journals.plos.org/plosone/s/file?id=wjVg/PLOSOne_formatting_sample_main_body.pdf>

and

<https://journals.plos.org/plosone/s/file?id=ba62/PLOSOne_formatting_sample_title_authors_affiliations.pdf>

**Response:** Thank you for the comment. We carefully reviewed the manuscript and revised it in accordance with the PLOS ONE style requirements, including formatting, manuscript organization, title page, headings, tables, figures, references, and file naming conventions.

2. Please request copy editing

**Response:** Thank you for the comment. We carefully reviewed the manuscript.

3. Thank you for stating the following in the Competing Interests section:

“The authors have declared that no competing interests exist.”

We note that one or more of the authors are employed by a commercial company: “Master Araştırma Eğitim ve Danışmanlık Hizmetleri Ltd.”

**Response:** Thank you for the comment. We have revised the Competing Interests Statement to explicitly declare the commercial affiliation of one of the authors with Master Araştırma Eğitim ve Danışmanlık Hizmetleri Ltd. Şti. We also clarified that the company had no role in the study design, data collection and analysis, decision to publish, or preparation of the manuscript. Furthermore, we confirmed that this commercial affiliation does not alter our adherence to PLOS ONE policies on sharing data and materials, as requested.

1. Please provide an amended Funding Statement declaring this commercial affiliation, as well as a statement regarding the Role of Funders in your study. If the funding organization did not play a role in the study design, data collection and analysis, decision to publish, or preparation of the manuscript and only provided financial support in the form of authors' salaries and/or research materials, please review your statements relating to the author contributions, and ensure you have specifically and accurately indicated the role(s) that these authors had in your study. You can update author roles in the Author Contributions section of the online submission form.

Please also include the following statement within your amended Funding Statement.

“The funder provided support in the form of salaries for authors [insert relevant initials], but did not have any additional role in the study design, data collection and analysis, decision to publish, or preparation of the manuscript. The specific roles of these authors are articulated in the ‘author contributions’ section.”

If your commercial affiliation did play a role in your study, please state and explain this role within your updated Funding Statement.

**Response:** Thank you for the comment. We have revised the Funding Statement to clarify the commercial affiliation of one of the authors with Master Araştırma Eğitim ve Danışmanlık Hizmetleri Ltd. Şti. We confirm that the company did not provide any financial support for this study and had no role in the study design, data collection and analysis, decision to publish, or preparation of the manuscript.

2. Please also provide an updated Competing Interests Statement declaring this commercial affiliation along with any other relevant declarations relating to employment, consultancy, patents, products in development, or marketed products, etc.

Within your Competing Interests Statement, please confirm that this commercial affiliation does not alter your adherence to all PLOS ONE policies on sharing data and materials by including the following statement: "This does not alter our adherence to PLOS ONE policies on sharing data and materials.” (as detailed online in our guide for authors http://journals.plos.org/plosone/s/competing-interests) . If this adherence statement is not accurate and there are restrictions on sharing of data and/or materials, please state these. Please note that we cannot proceed with consideration of your article until this information has been declared.

**Response:** Thank you for the comment. We have revised the Competing Interests Statement to explicitly declare the commercial affiliation of one of the authors with Master Araştırma Eğitim ve Danışmanlık Hizmetleri Ltd. Şti. We also clarified that this affiliation did not influence the study design, data collection and analysis, decision to publish, or preparation of the manuscript. Furthermore, we added the requested statement confirming that this commercial affiliation does not alter our adherence to PLOS ONE policies on sharing data and materials.

3. Please include both an updated Funding Statement and Competing Interests Statement in your cover letter. We will change the online submission form on your behalf.

**Response:** Thank you for the comment. We have updated our Cover Letter to include both the revised Funding Statement and Competing Interests Statement. The Funding Statement clarifies that the study received no specific funding and that the commercial affiliation had no role in the study design, data collection and analysis, decision to publish, or preparation of the manuscript. The Competing Interests Statement explicitly declares the commercial affiliation, confirms that it does not constitute a competing interest, and states that it does not alter our adherence to PLOS ONE policies on sharing data and materials.

4. If the reviewer comments include a recommendation to cite specific previously published works, please review and evaluate these publications to determine whether they are relevant and should be cited. There is no requirement to cite these works unless the editor has indicated otherwise.

**Response:** Thank you for the comment. The reviewer did not recommend any specific previously published works for citation. Therefore, no additional references were added in response to this request.

**Comments to the Author**

1. Is the manuscript technically sound, and do the data support the conclusions?

The manuscript must describe a technically sound piece of scientific research with data that supports the conclusions. Experiments must have been conducted rigorously, with appropriate controls, replication, and sample sizes. The conclusions must be drawn appropriately based on the data presented.

Reviewer #1: Partly

**Response:** Thank you for the comment. We have carefully revised the manuscript to address all methodological and interpretative concerns raised during the review process. Specifically, we strengthened the theoretical framework, expanded the methodological description, added hypotheses, reported additional model diagnostics and odds ratios with confidence intervals, and revised the discussion and interpretation of the findings.

2. Has the statistical analysis been performed appropriately and rigorously?

Reviewer #1: No

**Response:** Thank you for the comment. We have substantially revised the statistical analysis section to improve its rigor and transparency. Specifically, we expanded the description of the binary logistic regression model, clarified the rationale for using average marginal effects, reported additional model fit statistics (likelihood ratio test, McFadden's pseudo R², Nagelkerke R², and classification accuracy), assessed multicollinearity using VIF and tolerance values, and presented odds ratios with 95% confidence intervals.

3. Have the authors made all data underlying the findings in their manuscript fully available?

The PLOS Data policy requires authors to make all data underlying the findings described in their manuscript fully available without restriction, with rare exception (please refer to the Data Availability Statement in the manuscript PDF file). The data should be provided as part of the manuscript or its supporting information, or deposited to a public repository. For example, in addition to summary statistics, the data points behind means, medians and variance measures should be available. If there are restrictions on publicly sharing data—e.g. participant privacy or use of data from a third party—those must be specified.

Reviewer #1: Yes

**Response:** Thank you for the comment.

4. Is the manuscript presented in an intelligible fashion and written in standard English?

PLOS ONE does not copyedit accepted manuscripts, so the language in submitted articles must be clear, correct, and unambiguous. Any typographical or grammatical errors should be corrected at revision, so please note any specific errors here.

Reviewer #1: Yes

**Response:** Thank you for the comment.

**5. Review Comments to the Author**

**Reviewer #1:**

This manuscript investigates the socioeconomic determinants of online food ordering behavior in Türkiye using nationally representative microdata from the 2023 Turkish Statistical Institute (TurkStat) Household Information Technologies Usage Survey.

The researchers have clearly explained the importance of the topic in the introduction and used current literature. However, the main claim in the article is: "This is the first study in Türkiye using national microdata." It is insufficient to fill a scientific gap. For example, what are the shortcomings of existing studies? What theoretical gap does this study fill? Why is Turkey specifically being considered? What is Türkiye's importance in this regard on an international platform? The literature repeteadly refers to established frameworks such as TAM (Technology Acceptance Model) and TPB (Theory of Planned Behavior) . However, these theories are not incorporated into the conceptualization of the study. Consequently, the analysis remains largely descriptive rather than theory-driven. The hypotheses of the article are not included. While PLOS ONE does not require hypothesis-driven research, clearly stated hypotheses would strengthen the scientific rationale and improve the coherence between literature review and empirical analysis.

**Response:** Thank you for the comment. We substantially revised the Introduction and Literature Review to strengthen the scientific rationale of the study. Specifically, we clarified the research gap by explaining the limitations of previous studies and highlighting the need for nationally representative evidence on actual online food ordering behaviour. We also expanded the discussion of Türkiye's relevance as an emerging digital market with considerable regional socioeconomic disparities. Furthermore, we incorporated the Theory of Planned Behaviour (TPB) as the conceptual framework, discussed the Technology Acceptance Model (TAM) within the literature, and formulated eleven research hypotheses (H1–H11) to establish a clearer theoretical foundation and improve the coherence between the literature review and the empirical analysis.

The hypotheses of the article are not included.

**Response:** Thank you for the comment. To strengthen the theoretical foundation and improve the coherence between the literature review and the empirical analysis, we formulated and explicitly stated eleven research hypotheses (H1–H11) based on the existing literature and the Theory of Planned Behaviour (TPB). These hypotheses have been incorporated into the revised manuscript in the Literature Review section.

The methods section of the study does not include the logistic regression equation. Since model fit statistics are not provided, the model's performance cannot be evaluated in this form. The authors state that "multicollinearity was not detected". Although VIF values are presented, additional information such as Tolerance values and Mean VIF should be reported.

**Response:** Thank you for the comment. We have substantially revised the Statistical Analysis section. Specifically, we included the binary logistic regression equations and provided a more detailed description of the model. In addition, we reported model fit statistics, including the likelihood ratio (LR) chi-square test, McFadden's pseudo R², Cragg and Uhler (Nagelkerke) R², and classification accuracy to facilitate the evaluation of model performance. Multicollinearity diagnostics were expanded by reporting the Variance Inflation Factor (VIF) values.

One of the most concerning results in the article is the negative coefficeient for "Information search about goods/services" variable. The model suggests that individuals who search online for information about goods and services are less likely to order food online. This result contradicts both the literature and logic. The authors acknowledge the inconsistency but provide no convincing explanation. In the Discussion section, the authors only state, "This result contradicts previous studies." However, perhaps the data could be reviewed again.

**Response:** Thank you for the comment. We carefully re-examined the data and re-estimated the regression model to verify this unexpected finding. The negative association remained unchanged. We therefore expanded the Discussion substantially to provide a more comprehensive interpretation. Specifically, we clarified that the TurkStat variable measures general online information searching for goods and services rather than food-related information seeking. Consequently, individuals may search for products such as electronics, clothing, or travel services without subsequently ordering food online. We also noted that the association was only marginally significant and should therefore be interpreted with caution. Furthermore, we discussed this finding within the framework of the Theory of Planned Behaviour, emphasizing that information search alone may not necessarily translate into behavioural intention or actual purchasing behaviour because this relationship is influenced by attitudes, perceived behavioural control, and contextual factors.

The study does not explain why the 12 regions were reduced to 3.

**Response:** Thank you for the comment. We have clarified the rationale for grouping Türkiye's 12 NUTS Level-1 regions into three broader geographical regions. Specifically, this classification was adopted to capture broad regional socioeconomic and digital development differences while improving statistical power, model parsimony, and the interpretability of the regression model.

Logistic regression was used in the study, but no odds ratio was given. Some methodological errors are noticeable in the study. For example, the ME (Mean Time) for those aged 65+ was determined as -1.260. Theoretically, the marginal effect is expected to be between -1 and +1. It is recommended that this be reviewed. Some formatting inconsistencies were identified in the tables. For example, Table * "37.03" is written.

**Response:** Thank you for the comment. The reported estimates were obtained using the margins, eydx(*) command in Stata. Therefore, some estimates may exceed the conventional −1 to +1 range because they represent the relative proportional change in the predicted probability associated with changes in the independent variables. To avoid potential misunderstanding, we revised the manuscript to clarify the estimation approach, the interpretation of the reported effects, and the distinction between semi-elasticities and conventional average marginal effects. Additional methodological clarification was also added to the Methods section.

The discussion section lacks commentary. The authors did not provide any explanation, stating, "Our findings are consistent with previous studies." The discussion should provide deeper explanations regarding the mechanisms underlying the observed relationships.. The negative effect of the "information search" variable is a noteworthy finding, but they did not conduct a detailed analysis of it.

**Response:** Thank you for the comment. We substantially revised the Discussion section to move beyond merely comparing our findings with previous studies. For each major determinant, we now provide possible behavioural, socioeconomic, and technological mechanisms underlying the observed associations. Specifically, we expanded the interpretation of gender, age, education, income, occupation, digital device ownership, regional disparities, social media use, and household size by explaining how differences in digital literacy, technology access, time constraints, purchasing power, occupational routines, regional infrastructure, and household consumption patterns may influence online food ordering behaviour. We also added a detailed discussion of the unexpected negative association between online information searching for goods and services and online food ordering. We clarified that the TurkStat variable measures general information searching for goods and services rather than food-specific information seeking. Therefore, individuals may search for electronics, clothing, travel, or other services without subsequently ordering food online. We further explained that online information search may involve price comparison or information gathering followed by an offline purchase and may not necessarily translate into actual online purchasing behaviour. From the perspective of the Theory of Planned Behaviour, information search alone may be insufficient to generate behavioural intention or actual behaviour, since this transition is also shaped by attitudes, perceived behavioural control, and contextual factors.

The study also failed to address limitations.

**Response:** Thank you for the comment. We have added a dedicated Limitations section to the revised manuscript. This section now explicitly discusses the main limitations of the study, including the cross-sectional design, the use of self-reported data, the lack of detailed information on online food ordering behaviour, and the measurement limitations of some variables.
