# Supplementary material for: Digital food delivery services in an emerging market: socioeconomic determinants and implications for service adoption in Türkiye
Source: PLoS One. 2026 Nov 12;21(11):e0349630. doi: 10.1371/journal.pone.0349630 (PMC13759073; doi:10.1371/journal.pone.0349630)
Supplement: Supplementary file 2 [file pone.0349630.s003.docx]

**Dear Editor and Reviewers,**

Thank you very much for your comments concerning our manuscript titled “Digital food delivery services in an emerging market: socioeconomic determinants and implications for service adoption in Türkiye”. These comments have been very helpful in reviewing and improving our manuscript. We have carefully revising these instructive comments and made corrections that we hope will be approved. The revised parts are highlighted in red in the main paper.

The corrections to the manuscript and responses to the reviewer’s comments are as following:

Regards,

Authors

**Comments to the Author**

1. If the authors have adequately addressed your comments raised in a previous round of review and you feel that this manuscript is now acceptable for publication, you may indicate that here to bypass the “Comments to the Author” section, enter your conflict of interest statement in the “Confidential to Editor” section, and submit your "Accept" recommendation.

Reviewer #1: (No Response)

2. Is the manuscript technically sound, and do the data support the conclusions?

The manuscript must describe a technically sound piece of scientific research with data that supports the conclusions. Experiments must have been conducted rigorously, with appropriate controls, replication, and sample sizes. The conclusions must be drawn appropriately based on the data presented.

Reviewer #1: Yes

**Response:** Thank you for the comment.

3. Has the statistical analysis been performed appropriately and rigorously?

Reviewer #1: Yes

**Response:** Thank you for the comment.

4. Have the authors made all data underlying the findings in their manuscript fully available?

The PLOS Data policy requires authors to make all data underlying the findings described in their manuscript fully available without restriction, with rare exception (please refer to the Data Availability Statement in the manuscript PDF file). The data should be provided as part of the manuscript or its supporting information, or deposited to a public repository. For example, in addition to summary statistics, the data points behind means, medians and variance measures should be available. If there are restrictions on publicly sharing data—e.g. participant privacy or use of data from a third party—those must be specified.

Reviewer #1: Yes

**Response:** Thank you for the comment.

5. Is the manuscript presented in an intelligible fashion and written in standard English?

PLOS ONE does not copyedit accepted manuscripts, so the language in submitted articles must be clear, correct, and unambiguous. Any typographical or grammatical errors should be corrected at revision, so please note any specific errors here.

Reviewer #1: Yes

**Response:** Thank you for the comment.

**6. Review Comments to the Author**

**Reviewer #1:**

After carefully reviewing the revised manuscript and the authors’ responses to my comments, I acknowledge that the manuscript has been substantially improved and that most of my previous concerns have been addressed. However, I still have several concerns that should be addressed before the manuscript can be considered for publication. Most importantly, the interpretation and presentation of the reported marginal effects require further clarification. The use of the margins, eydx(*) command appears to produce semi-elasticity-type estimates rather than conventional average marginal effects, yet these estimates are presented as “marginal effects” and interpreted as percentage changes in probability. In particular, the reported value of −1.260 for individuals aged 65+ remains potentially misleading and should be re-examined, by providing a clear and technically accurate explanation of the current estimates and their interpretation. In addition, there appears to be an inconsistency between the statistical significance reported for the “information search about goods/services” variable in the regression results and that reported in the marginal-effects table, which should be carefully checked and corrected. Additionally a typographical error in the logistic regression equation (β0 appears to be repeated where β1 should be used) and an inaccurate statement describing logistic regression coefficients as changes in a “z-score”; this should be corrected. Thank you.

**Response:** Thank you for the comment. We agree that the previous presentation of the marginal effects required clarification and have revised both the analysis and the manuscript accordingly. In the previous version, the estimates were obtained using the Stata command margins, eydx(*), which produces semi-elasticity-type estimates rather than conventional average marginal effects (AMEs). To eliminate any potential ambiguity and to provide directly interpretable probability-based effects, we re-estimated the marginal effects using the conventional average marginal effects approach (margins, dydx(*)). Accordingly, Table 5 has been completely updated with the newly calculated AMEs, and all corresponding interpretations in the Results section have been revised. The effects are now expressed as percentage-point differences in predicted probability, rather than percentage changes in probability. For example, the previously reported value of −1.260 for individuals aged 65 years and over has been replaced by an AME of −0.350, indicating that, compared with individuals aged 16–24 years, those aged 65 years and over have a 35.0-percentage-point lower predicted probability of ordering food online, holding the other variables constant. We carefully rechecked the statistical significance of the “information search about goods/services” variable. In the logistic regression coefficient results, this variable is significant at the 10% level, whereas its newly calculated average marginal effect is significant at the 5% level. We verified these results against the statistical output and retained the corresponding significance levels in the respective tables. To prevent misinterpretation, we have also explicitly acknowledged this difference in the Discussion and emphasized that this finding should be interpreted cautiously, particularly because the information-search variable is highly unbalanced, with very few observations in the reference category. We corrected the typographical error in the logistic regression equation, where β₀ had been inadvertently repeated instead of β₁. The equation has now been corrected to accurately represent the logistic regression specification. We corrected the inaccurate description of logistic regression coefficients as changes in a “z-score.” The revised Methods section now correctly states that the estimated coefficients represent changes in the log-odds of the outcome associated with a one-unit change in an independent variable, holding the other variables constant. We also clarify that the sign of the coefficient indicates the direction of the association, whereas its magnitude is not directly interpretable as a change in probability.
